# Cesarean Scar Pregnancy Case Report in a Grade 2 Maternity and Review of the Literature

**DOI:** 10.3390/reports8040267

**Published:** 2025-12-15

**Authors:** Muntean Mihai, Gliga Cosma Marius, Sasaran Vladut, Mărginean Claudiu

**Affiliations:** Department of Obstetrics and Gynecology, University of Medicine Pharmacy Science and Technology George Emil Palade of Târgu Mureș, 540142 Târgu Mureș, Romania; munteanmihai@yahoo.com (M.M.); m11gliga@yahoo.com (G.C.M.); marginean.claudiu@gmail.com (M.C.)

**Keywords:** cesarean scar, cesarean scar pregnancy, pregnancy, niche

## Abstract

**Background and Clinical Significance**: Cesarean scar pregnancy (CSP) is a rare complication that can occur after cesarean delivery, potentially exposing the patient to severe and life-threatening complications. This case report aimed to illustrate the evolution of CSP cases following initial conservative surgical treatment. **Case Presentation:** We present two cases involving pregnant women aged 29 and 36 years, both diagnosed with type 2 CSP based on ultrasound findings between 6 and 8 weeks of gestation. In these cases, we initially performed an aspirative curettage after administering systemic Methotrexate (MTX) or Mifepristone. Unfortunately, both patients experienced severe bleeding during the aspirative curettage, which necessitated emergency abdominal hysterectomy along with bilateral salpingectomy. Additionally, we provide an updated review of the related literature. **Conclusions:** For optimal outcomes, treatment must be tailored to various factors, including gestational age at diagnosis, gestational sac size, anterior myometrial thickness, and the presence of embryonic cardiac activity.

## 1. Introduction and Clinical Significance

In recent decades, the global incidence of cesarean section rates increased from 7% in 1990 to 21% [1] and in some countries, even to 54.6% [2]. In addition, there are some benefits for women regarding cesarean delivery, like a reduced rate of pelvic organ prolapse and urinary incontinence compared with vaginal delivery [3], there are risks for long-term future health for the women, like abnormal placentation, uterine rupture during a subsequent pregnancy, and some gynecological complications like adhesions, chronic pelvic pain, infertility, irregular bleeding, painful menses and endometriosis [4].

One of the rare complications after cesarean section is a pregnancy implanted in the scar from a previous cesarean section. Its incidence is difficult to estimate, but some studies have found that the incidence of scar pregnancy is between 1/1800 and 1/2500 of patients who gave birth by cesarean section [5]. There are two types of cesarean scar pregnancy (CSP): in type 1, the implantation occurs “on the scar” with measurable myometrial thickness (≥2–3 mm) between the gestational sac and the bladder, and type 2, with the gestational sac implanted “in the niche” with thin myometrium (<2 mm) between the gestational sac and the bladder [5]. The symptoms of the patient with CSP are variable; most of the time, it is asymptomatic, but vaginal bleeding in the first trimester and lower abdominal pain may be present. The diagnosis of CSP is usually made by transvaginal ultrasound; magnetic resonance imaging (MRI) is rarely necessary for diagnosis [5]. Accurate diagnosis of a CSP within the first 7 weeks of pregnancy, followed by timely termination, is essential. This approach helps prevent serious complications associated with ongoing CSP, such as placenta accreta spectrum, uterine rupture, heavy vaginal bleeding, and shock, which may ultimately necessitate an emergency hysterectomy [5]. In this paper, we present two cases of CSP managed in our Level 2 clinic, along with a review of the current evidence in the literature.

## 2. Case Presentation

**Figure 1:**
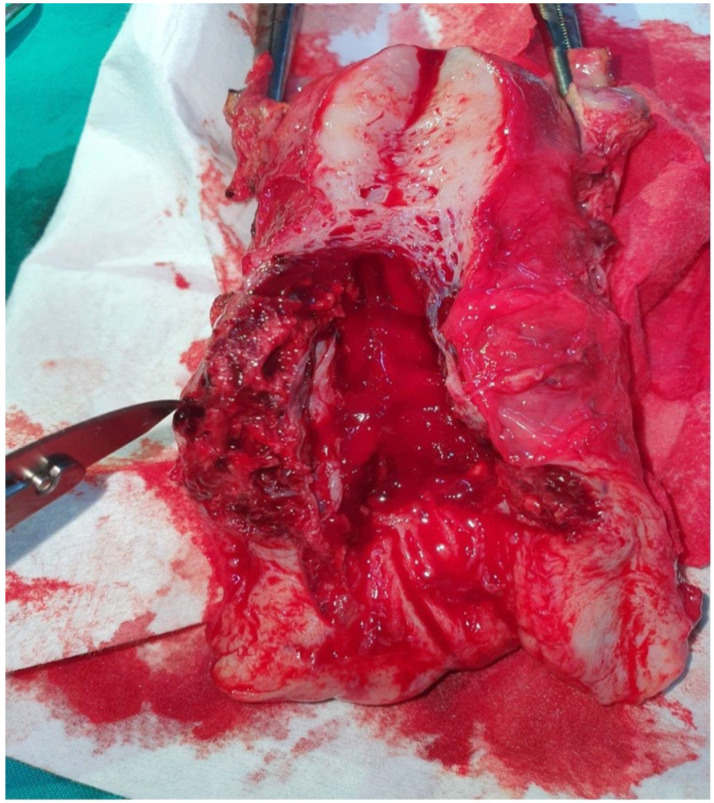
Uterus with a cesarean scar pregnancy after total hysterectomy with bilateral salpingectomy.

Case 1: A 36-year-old woman, gravida 3, para 1, with a history of one previous caesarean section (three years prior), presented to our clinic at eight weeks of gestation due to light vaginal bleeding. A transvaginal ultrasound examination revealed a gestational sac located in the caesarean scar, with a thin myometrium (1–2 mm) adjacent to the urinary bladder. An embryo was identified with a crown-rump length of 15 mm, but there was no evidence of cardiac activity. The patient had no significant medical history and had not undergone any previous surgical procedures. We did not have serum β-human chorionic gonadotropin (β-hCG) levels available at that time. On physical examination, the findings were normal for cardiovascular, respiratory, and abdominal assessments. The medical team counseled the patient about the potential severity of her condition and discussed all possible life-threatening complications that could arise. We also reviewed all available medical and surgical management options in detail. Initially, after counseling, the patient chose to pursue conservative treatment. A dose of 200 mg of Mifepristone was administered orally. However, after one day, the patient experienced significant vaginal bleeding and was admitted for further care. All preoperative tests were found to be within normal limits. Subsequently, a uterine aspiration curettage was tried under ultrasound guidance as a method of conservative management. Due to significant bleeding during the suction curettage procedure, an emergency total hysterectomy with bilateral salpingectomy was performed. After the surgery, anemia was treated by administering isogroup isoRh blood. The patient was discharged in good overall condition on the fifth postoperative day. The pathological examination of the surgical specimen revealed a pregnancy at the cesarean scar without myometrial invasion (Figure 1).

**Figure 2:**
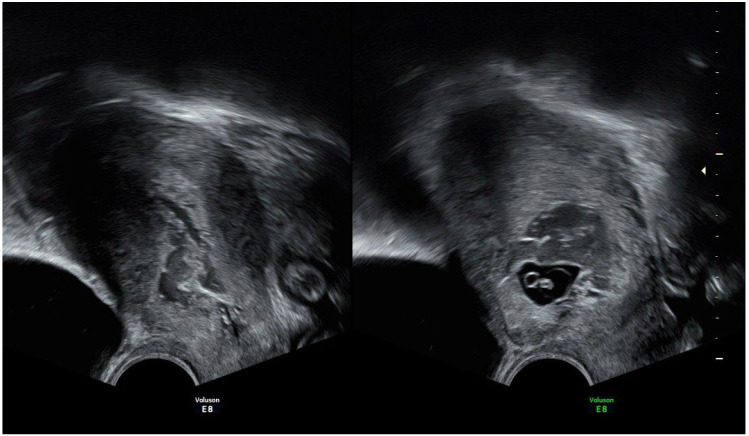
Ultrasound images of the uterus showing a gestational sac in the cesarean scar, with an empty uterine cavity and an empty cervical canal.

Case 2: A 29-year-old woman, gravida 3, para 2, with a history of two previous caesarean sections (one year prior), presented to our clinic at six weeks of gestation due to lower abdominal pain. A transvaginal ultrasound examination revealed a gestational sac located in the caesarean scar, with a thin myometrium (2.1 mm) adjacent to the urinary bladder (Figure 2). An embryo with a CRL corresponding to 6 weeks plus 0 days was identified, but there was no evidence of cardiac activity. The patient had no significant medical history and had not undergone any previous surgical procedures. We did not have β-hCG levels available at that time. The physical examination findings for the cardiovascular, respiratory, and abdominal assessments were all normal. The medical team counseled the patient about the potential severity of her condition and discussed all possible life-threatening complications that could arise. We also reviewed all available medical and surgical management options in detail. Patient opted for conservative management and received a dose of 50 mg/m^2^ of Methotrexate. The patient was admitted to the hospital 3 days after Methotrexate administration for uterine aspiration curettage. Preoperative tests were within normal limits. Suction curettage was performed under ultrasound guidance, but due to significant bleeding during the suction curettage procedure and signs of intra-abdominal bleeding from uterine perforation in the cesarean scar region of the uterus, an emergency total hysterectomy with bilateral salpingectomy was performed. After the surgery, anemia was treated by administering isogroup isoRh blood. The patient was discharged in good overall condition on the fourth day after surgery. The pathological examination of the surgical specimen revealed a pregnancy at the cesarean scar with signs of myometrial invasion (placenta increta). Reviewing ultrasound pictures of the uterus prior to cesarean scar pregnancy, the patient did not have signs of a niche in the cesarean scar.

Patients’ demographic, clinical characteristics, and outcomes are summarized in Table 1.

## 3. Discussion

One rare complication that can occur after a cesarean section is a cesarean scar pregnancy. Although the exact incidence is hard to determine, some studies suggest that the occurrence of scar pregnancies ranges from 1/1800 to 1/2500 among women who have given birth via cesarean section [5]. In our level 2 maternity hospital, the cesarean section rate has remained stable over the last 10 years, averaging 40%. Over the past 10 years, we have documented 2 cases of cesarean scar pregnancy, resulting in an incidence rate of 1/2914 cesarean sections, which is lower than what is described in the literature. The lower incidence of CSP observed in our clinic may be due to the lower parity among patients with a history of cesarean births over the past 10 years. Additionally, it is possible that not all patients with CSP, who had their cesarean sections performed in our clinic, sought diagnosis and treatment for their condition here.

CSP occurs when a blastocyst implants in a tract on the uterine scar or in the “niche” left by a previous cesarean delivery incision. Healing defects in the uterine wall at the cesarean scar level can lead to gynecologic complications such as abnormal vaginal bleeding, chronic pelvic pain, and infertility. Additionally, they may result in CSP, uterine rupture, and the placenta accreta spectrum in subsequent pregnancies [6].

In their review, Verberkt et al. [7] found that histopathological findings associated with “niche” development were necrosis, inflammation, and insufficient approximation.

Regarding patient-related risk factors associated with “niche” development, they found that multiple cesarean sections (CS), higher body mass index (BMI), and smoking were associated with “niche” development. Labor-related factors identified included CS performed before the onset of labor, extended cervical dilation, premature rupture of membranes (PROM), and the fetal presenting part being positioned below the pelvic inlet during the CS.

In their study on the factors influencing the healing process after a cesarean section, Budny-Winska et al. [8] found no correlation between maternal or gestational age at delivery, the presence of medical complications during pregnancy (such as diabetes mellitus, gestational hypertension, and hypothyroidism), cervical canal colonization, and characteristics of a niche 6 to 9 weeks after the cesarean section. Pomorski et al. [9] conducted a study on a group of women with a history of one cesarean section. They found that maternal age and uterine retroflection were linked to a greater depth of the cesarean “niche”. Additionally, women without a scar defect exhibited significantly higher residual myometrial thickness values, lower newborn birth weights, and a higher position of the scar above the internal cervical os.

Regarding the state of the cesarean scar prior to the CSP in our cases, we had ultrasound images related to cesarean scar before pregnancy with CSP only in case 2, which showed no ultrasound signs of a “niche”.

The impact of uterine suture techniques during cesarean sections on the risk of developing a “niche” has been a topic of debate in the literature. Some experts like Genovese et al. [6] found that a continuous two-layer suture is more effective than a monoplane suture, while others like Verberkt et al. [10] have found no significant advantage of using a two-layer suture over a monoplane suture. Verberkt et al. [7] found that preventive measures for niche formation include using full-thickness closure of the myometrium with non-locking sutures and avoiding a low incision in cases of increased cervical dilation. In our clinic, we use a continuous monoplane non-locking suture technique for the uterine wall closure during cesarean sections. While we lack data on the long-term evolution of patients who received continuous monoplane sutures compared to those who received continuous two-layer sutures during cesarean sections, the observed incidence of CSP over time in our clinic is slightly lower than what is reported in the literature. This suggests that using monoplane uterine sutures during cesarean sections remains a viable option.

Most of the time, patients with CSP are asymptomatic, but vaginal bleeding in the first trimester and lower abdominal pain may be present [11]. A diagnosis of CSP at a gestational age of less than 9 weeks is associated with a significantly lower risk of maternal complications, according study by Timor-Tritsch [12]. This finding supports the idea of implementing universal screening for CSP in women who have a history of cesarean delivery. A late presentation to the hospital during the first trimester, particularly when there are no symptoms present, can lead to difficulties in obtaining an accurate diagnosis. This delay may be associated with the development of placenta accreta spectrum later in pregnancy, potentially leading to an emergency hysterectomy at birth, fetal death, preterm birth, hemorrhagic morbidity, and surgical complications [13,14]. In our two cases presented above, the symptoms included vaginal bleeding in the first case and lower abdominal pain in the second, and gestational age at diagnosis was below 9 weeks of gestation.

In our cases, the diagnosis CSP was made using transvaginal ultrasonography at 8 weeks of gestation and at 6 weeks of gestation. In both cases, we observed an empty uterine cavity and cervical canal, with a gestational sac located in the cesarean scar (type 2 CSP).

Timor-Tritsch et al. [11] recommend considering a diagnosis of a CSP in cases where there is a history of a previous cesarean section and a low, anterior gestational sac, especially in an anteverted retroflexed uterus, regardless of whether there is heart activity. This diagnosis should be made unless proven otherwise before any surgical intervention takes place. The initial ultrasound, performed between 5 and 7 weeks, is essential, as many CSPs are frequently misdiagnosed as threatened abortions, miscarriages, cervical pregnancies, or low implantation of a normal intrauterine pregnancy [11,15]. Such misdiagnoses can result in curettage for a presumed failed pregnancy, leading to significant bleeding, the need for emergency surgical interventions, and, in some cases, hysterectomy [11]. The Society for Maternal-Fetal Medicine proposed some ultrasonographic criteria for diagnosing CSP: (1) an empty uterine cavity and endocervix; (2) placenta, gestational sac, or both embedded in the hysterotomy scar; (3) a triangular (at 8 weeks of gestation) or rounded or oval (at >8 weeks of gestation) gestational sac that fills the scar “niche” (4), a thin (1–3 mm) or an absent myometrial layer between the gestational sac and bladder; (5) a rich vascular pattern at or in the area of a cesarean scar; and (6) an embryonic pole, yolk sac, or both, with or without embryonic cardiac activity [15]. When ultrasonography fails to provide sufficient images for a definitive diagnosis, pelvic MRI can be utilized as an alternative [15].

Due to the significant risk of severe complications associated with ongoing CSP, including placenta accreta spectrum, uterine rupture, heavy vaginal bleeding, and shock, which may ultimately necessitate an emergency hysterectomy, the Society for Maternal-Fetal Medicine recommends against expectant management [12,15]. In both cases, we combined drug treatment and aspiration curettage of the gestational sac as a conservative treatment option. However, due to complications that occurred during the curettage, total hysterectomy with bilateral salpingectomy was ultimately performed in both cases.

Both medical and surgical methods for treating CSP have been discussed in the literature.

In their systematic review on the systemic administration of methotrexate or mifepristone for the medical treatment of CSP, Stabile et al. [16] concluded that this approach has a success rate of 71.4%. The treatment seems to be most effective when the levels of β-human chorionic gonadotropin (β-hCG) are below 5,000 mIU/mL and when the gestational sac measures less than 20 mm. Additionally, the absence of a fetal heartbeat appears to be a positive prognostic factor for achieving a favorable outcome.

In their study, Cagli et al. [17] administered 50 mg of MTX through transvaginal ultrasound-guided single-dose local treatment for CSP. The median gestational age at the time of diagnosis was 7 weeks and 2 days. The mean β-hCG was 31,345 ± 37,838 mIU/mL, with a range of 113 to 233,835 mIU/mL. A total of fifty-four patients were effectively treated with local MTX therapy. The average interval between the initial MTX injection and the normalization of β-hCG levels was 55.2 ± 41.0 days. Notably, none of the patients required surgical intervention. The authors concluded that this method is an effective, safe, and fertility-preserving treatment option for CSP.

Timor-Tritsch et al. [18] described in their article the use of pressure balloons as a minimally invasive procedure for the management of CSP. The double-cervical office-based balloon catheter has distinct advantages for managing early first-trimester CSPs up to 8 weeks of gestation. It can be used as a primary treatment option or as an adjunct to other treatments. This method provides a tamponade effect, which helps prevent and treat potential bleeding complications.

The literature describes a variety of minimally invasive approaches for treating CSP. The choice of method depends on factors such as the severity of the patient’s symptoms, the availability of resources, and the surgical skills of the medical team. Some of the approaches include: ultrasound-guided curettage, curettage combined with MTX, local administration of MTX accompanied by hysteroscopy, laparoscopic monitored curettage, laparoscopic resection of CSP, transvaginal resection, uterine artery embolization (UAE) combined with dilatation and curettage, UAE alongside hysteroscopy, hysteroscopic resection of CSP [19,20,21,22,23].

Li et al. [23] concluded in their meta-analysis that the curative effect of UAE combined with dilatation and curettage (D&C) is similar to that of abdominal and vaginal resection surgeries, but is inferior to laparoscopic resection surgery. In their systematic review, Birch et al. [20] concluded that the use of dilatation and curettage as a treatment approach for CSP is associated with a significant complication rate of 21%, as well as a requirement for additional interventions in 52% of cases.

In their study, Xu et al. [21] treated 117 patients with CSP based on the specific type of CSP. Patients with type 1 CSP underwent ultrasound-guided curettage, while those with type 2 CSP received either laparoscopic-guided curettage or laparoscopic resection of the CSP. The researchers concluded that all these methods are safe and effective. During the postoperative follow-up, 42.1% of the patients became pregnant again, and the recurrence rate of CSP was 4.1%.

In a study involving 54 patients with CSP, Di Spiezio et al. [22] compared two treatment methods: hysteroscopic resection and ultrasound-guided dilation and evacuation. The procedures were performed, on average, three days after the intramuscular administration of 50 mg/m^2^ of methotrexate (MTX). The results indicated that hysteroscopic resection was associated with a higher success rate in treating CSPs compared to ultrasound-guided dilation and evacuation.

Zhang et al. [24] in their study on 112 patients with CSP treated with ultrasound-guided curettage and hysteroscopic resection without MTX or UAE before the procedure, found that CSP patients with more than 52.5 days of amenorrhea, a gestational sac diameter greater than 3.25 cm, and a myometrial thickness less than 2.05 mm, laparoscopic repair of the uterine defect may be necessary in addition to curettage and hysteroscopy. Yang et al. [25] conducted a study involving 1637 patients with CSP to investigate the factors associated with treatment failure under various strategies. The results showed no significant difference in the failure rates between ultrasound-guided evacuation and hysteroscopy-guided evacuation for CSP treatment, regardless of whether UAE was used as a pretreatment. The study found that a larger sac diameter, the presence of a fetal heartbeat, and a longer gestational age were all linked to a higher likelihood of initial treatment failure for CSP.

Ban et al. [26] developed a new clinical classification system for the treatment of CSP. This system consists of five classifications, which are based on myometrial thickness and the diameter of the gestational sac. The new classification system demonstrated an overall success rate of 97.5%, based on the results obtained from a cohort of 564 patients with CSP. The authors recommend different methods for managing CSP based on the thickness of the anterior myometrium (TAM) and the size of the gestational sac: 1. If the TAM is greater than 3 mm: suction curettage may be performed with or without hysteroscopy under ultrasound guidance. 2. If the TAM is between 1 mm and 3 mm, and the average diameter of the gestational sac is under 30 mm: suction curettage with hysteroscopy under ultrasound guidance is recommended, and if the average diameter of the gestational sac is greater than 30 mm: hysteroscopy with laparoscopic monitoring, excision, or transvaginal excision should be considered. 3. If the TAM is less than 1 mm and the average diameter of the gestational sac is under 50 mm: laparoscopic excision or transvaginal excision is advised, and if the gestational sac is greater than 50 mm: laparoscopic excision should be performed after UAE or a laparotomy may be necessary. This structured approach aims to tailor the management based on specific clinical criteria.

In cases where patients refused medical or surgical treatment and carried their pregnancies to term, a myometrial thickness of less than 3.3 mm around the gestational sac was associated with severe complications. These complications included significant intraoperative bleeding and severe forms of morbidly adherent placenta [27]. In their meta-analysis, Cali et al. [28] found that in cases of CSP without embryonic cardiac activity, expectant management may be reasonable given the low risk of maternal complications requiring intervention. However, close surveillance is recommended to prevent adverse maternal outcomes (Table 2).

In conclusion, we recommend that cases of CSP diagnosed before 8 weeks of gestation, particularly those with a TAM of less than 3 mm, be managed in facilities equipped to perform aspiration curettage or hysteroscopic resection under ultrasound guidance. This intervention should be carried out following the administration of systemic methotrexate (MTX). If conservative surgical treatment fails, a hysterectomy may be warranted.

## 4. Conclusions

Cesarean scar pregnancy is a rare complication that can occur after a cesarean delivery, potentially leading to serious complications for the patient. For the best possible outcome, treatment should be tailored to several factors, including gestational age at diagnosis, thickness of the anterior myometrium, size of the gestational sac, and presence of embryonic cardiac activity.

## Figures and Tables

**Table 1:** Patients’ demographic, clinical characteristics, and outcomes.

Characteristics	Case 1	Case 2
Age, years	36	29
Gestation	3	3
Parity	1	2
Previous cesarean sections	1	2
Gestational age, weeks	8	6
Previous medical or surgical pathology	no	no
β-hCG level	-	-
Symptoms	vaginal bleeding	lower abdominal pain
Medical treatment prior to surgical treatment	systemic mifepristone	systemic methotrexate
Effective suction curettage	no	no

Note: β-hCG - beta-human chorionic gonadotropin.

**Table 2:** Summary of the information from the above case reports and case series.

Author	Number of Patients	Gestational Age, Median, Weeks	Symptoms	MTX	Suction Curettage	Hysteroscopic Removal	Laparoscopic Removal	Expectant Management	Results
Cagli et al. [17]	56	7		local MTX (all patients)					normalization of β-hCG was in 55.2 ± 41.0 days
Casadio et al. [19]	1	9	Lower abdominal pain	local MTX		Hysteroscopic removal 6 weeks after MTX			effective
Xu et al. [21]	117				33 patients (21 with type 1 CSP)		84 patients (54 with type 2 CSP)		Both are effective
Di Spiezio et al. [22]	54	<8 weeks and 6 days		Systemic MTX (all patients)	27 patients	27 patients			Hysteroscopic removal superior to suction curettage
Zhang et al. [24]	112	7 weeks and 5 days			72 patients		42 patients		Both are effective
Fu et al. [27]	21							All patients	Low myometrial thickness associated with serious complications

Note: β-hCG: beta-human chorionic gonadotropin CSP: cesarean scar pregnancy, MTX: methotrexate.

## Data Availability

The original data presented in the study are included in the article, further inquiries can be directed to the corresponding author.
